# Avoiding biased exclusions in cluster trials

**DOI:** 10.1111/aogs.13776

**Published:** 2020-01-17

**Authors:** Jim Thornton, Helena E. Fadl, Kate F. Walker, David Torgerson

**Affiliations:** ^1^ Maternity Department Division of Obstetrics and Gynecology School of Clinical Sciences University of Nottingham City Hospital Nottingham UK; ^2^ Department of Obstetrics and Gynecology Faculty of Medicine and Health Örebro University Örebro Sweden; ^3^ Department of Obstetrics and Gynecology Queens Medical Centre University of Nottingham Nottingham UK; ^4^ York Trials Unit Department of Health Sciences University of York Heslington UK

Cluster trials are used increasingly to test interventions that need to be implemented at a group level, or that require testing on large populations, but they are notoriously susceptible to selection bias.

The problem also affects individually randomized trials, especially open ones, where participants may exclude themselves after they discover their treatment allocation, or if treatment takes time to organize, such that one group has less opportunity to receive it. In this type of trial, the problem is well recognized, and the solution is clear, namely to enter participants in the trial before randomization and to include all participants in their allocated group, whatever treatment they received, so called “analysis by intention‐to‐treat”.

For cluster trials, “analysis by intention‐to‐treat” requires first that all participants be recruited to the cluster before randomization or, if this is practically impossible, that recruitment post‐randomization is sufficiently automatic to rule out selective exclusion. Then second, the analysis must be conducted on the whole sample. Unfortunately, even major funders and high impact journals continue to miss this source of bias.

The stepped wedge PARROT trial reported in the *Lancet* in March^1^ is a recent example. Stepped wedge trials are a subtype of cluster trials, in which intervention centers act as their own controls, the control period precedes the intervention, and the intervention typically continues after the trial ends.^2^ The PARROT researchers were testing the effect of introducing a new test for preeclampsia, placental growth factor (PlGF).^3^ They randomized whole maternity units, each caring for many thousands of women over the study period, but analyzed only the minority who had suspected preeclampsia. On the face of it, maternal severe adverse outcomes were reduced (24/447 women in the concealed testing group vs 22/573 women in the revealed group, odds ratio [OR] 0.32, 95% CI 0.11‐0.96; *P* = 0.043), with no significant effect on perinatal adverse outcomes (86 revealed vs 63 concealed, OR 1.45, 95% CI 0.73‐2.90). The authors recommended adoption of PlGF testing.

However, the number of women identified with suspected preeclampsia had increased by 28%, from 447 to 573, during the period when the new PlGF test results were revealed and acted upon. As the recruitment periods were identical and birth rates had not altered, this is unlikely to have occurred by chance, and was probably due to inclusion of additional women with milder disease. No‐one can now identify the corresponding women during the control periods, but it is likely that they also had mild disease and good outcomes. If so, we can crudely model what the results would have been, had there been an additional 126 women and babies with good outcomes in the control period. The rate of severe adverse maternal outcomes would have become about 4% in both groups and the trend towards more adverse baby outcomes would have increased (15% vs 11%), favoring controls. Without access to the full data set we cannot estimate the statistical significance, but the conclusion of the trial might well have been the opposite!

The authors of the recent AFFIRM trial,^4^ also in the *Lancet*, avoided the problem. Hospitals were allocated at random to a package of care that encouraged mothers to be aware of, and report, alterations in fetal movements, and encouraged staff to respond appropriately. The authors reported all perinatal deaths occurring in each hospital over the relevant time periods, not just those in women who had experienced altered movements. The result was clear. The package was ineffective with an adjusted OR 0.98 (95% CI 0.83‐1.17) for perinatal mortality.

Similar problems have affected, and been avoided, in cluster trials of school‐based sex education interventions.

“Baby think it over”, a school‐based pregnancy prevention program in which teenage girls cared for a simulated infant, was evaluated in a cluster trial published in the *Lancet* in 2016.^5^ A higher proportion of the intervention group went on to have at least one birth as teenagers, 97/1267 (8%) vs 67/1567 (4%) control (RR 1.36, 95% CI 1.10‐1.67, *P* = 0.003) or at least one termination of pregnancy as the first pregnancy event (9% vs 6%). The headline results were that use of the infant simulator was harmful.

Unfortunately, only about half the girls in the intervention schools could be recruited because of the availability of school health nurses and infant simulators. This gave an opportunity for selection bias. Bolzern et al^6^ tested baseline factors for nominal statistical significance, and showed that some differences could not have occurred by chance; the intervention group was more socioeconomically disadvantaged (*P* = 0.000000000019) and had lower educational attainment (*P* = 0.0000000015). Teachers were probably recruiting girls who they thought were at higher risk, to the intervention groups. Analyzing pregnancies and abortions among all the girls in the intervention and control clusters, which would have avoided the problem, was not done.

In contrast, the investigators of SHARE, a cluster trial of school‐based peer‐led sex education published in the *BMJ*,^7^ did exactly that. Whole schools were allocated to intervention or control and every female member of the relevant class was followed up, whether or not they actually participated. There were no significant differences between the groups in registered conceptions per 1000 pupils (300 SHARE vs 274 control; difference 26, 95% CI −33 to 86), or in terminations per 1000 pupils (127 vs 112; difference 15, 95% CI −13 to 42) between ages 16 and 20 years. The results were disappointing for supporters of the intervention, but secure.

The present authors are involved in two ongoing cluster trials in obstetrics. JGT and KW with the GBS‐3 trial in the UK,^8^ and HF with the CDC4G trial in Sweden.^9^

GBS‐3 is a cluster trial testing the effect of adding routine Group B streptococcus screening at either 36 weeks or intrapartum, to the UK's present risk‐based screening policy. Eighty maternity units will be randomized. The primary end point, all‐cause early neonatal sepsis, will be measured from routine data for every birth within the clusters, whether or not the mother underwent screening.

CDC4G is a cluster trial testing the effect of lowering the threshold for diagnosing gestational diabetes. The primary outcome is the rate of fetal macrosomia. If this were measured among pregnancies where diabetes has been diagnosed, the result would be biased because the new threshold will almost certainly diagnose more women with diabetes. Instead it will be measured among all women delivering within the participating hospitals whatever their diabetes results.

So long as both trials analyze everyone in the randomized units, they should avoid the problems of PARROT and “Baby Think it Over”, and produce reliable results like AFFIRM and SHARE.
